# Dr. Lewis Blanchard

**Published:** 1913-12

**Authors:** 


Dr. Lewis Blanchard, Buffalo, 1866, died at his home in Edgewood, Ia., August 5.

